# Falling aid for reproductive, maternal, newborn and child health in the lead-up to the COVID-19 pandemic

**DOI:** 10.1136/bmjgh-2021-006089

**Published:** 2021-06-09

**Authors:** Catherine Pitt, David Bath, Peter Binyaruka, Josephine Borghi, Melisa Martinez-Alvarez

**Affiliations:** 1 Department of Global Health and Development, London School of Hygiene & Tropical Medicine, London, UK; 2 Department of Health System, Impact Evaluation and Policy, Ifakara Health Institute, Dar es Salaam, Tanzania; 3 Department of Global Health and Development, Medical Research Council Unit The Gambia at the London School of Hygiene & Tropical Medicine, Dakar, Senegal

**Keywords:** health systems, maternal health, paediatrics, public health

**Summary box**

- Greater investment in reproductive, maternal, newborn and child health (RMNCH) is needed to mitigate the negative effects of COVID-19 and avoid a reversal of recent gains in RMNCH coverage and outcomes.
- Aid for RMNCH as a whole fell by 6% between 2017 and 2018 and only increased by 2% in 2019; over the same 2-year period, aid for the reproductive health of non-pregnant women fell by 25%.
- Volatile and falling aid for RMNCH may have rendered RMNCH systems more fragile in the 2 years preceding the COVID-19 pandemic.
- We encourage everyone—academics, advocates and policy makers—to explore and exploit the Muskoka2 aid for RMNCH dataset and other aid datasets, as part of efforts to improve RMNCH outcomes.

## Introduction

Infectious disease epidemics pose a threat to reproductive, maternal, newborn and child health (RMNCH) both directly—by worsening women’s and children’s health outcomes—and indirectly—by reducing their access to services.^1–4^ Greater investment is therefore needed to mitigate the negative effects of COVID-19 and avoid a reversal of recent gains in RMNCH coverage and outcomes.^1^ However, COVID-19 has reduced household and government budgets,^5^ and there are concerns about the extent to which resources have been diverted away from RMNCH.^1^

Alongside domestic government financing and household out-of-pocket expenditure, aid plays a substantial role in funding RMNCH services in many countries. In 2018, for example, aid accounted for 25% of current health expenditure in the 45 least developed countries.^6^ Across the 23 countries with RMNCH expenditure estimates for 2018, aid accounted for a median of 22% of RMNCH expenditure, ranging from <1% in Namibia and the Seychelles up to 64% of child health and 84% of reproductive and maternal health expenditure in South Sudan.^6^ Yet, aid is volatile,^7^ subject to changing political priorities and not always responsive to needs.^6^ Global economic crises can lead to reduced aid levels and change the nature and sources of funding.^8^ Tracking disbursements of aid for RMNCH is therefore important for holding donors accountable for their commitments, including before, during and after crises.

## The Muskoka2 method for tracking aid for RMNCH

The Muskoka2 method was previously developed to track aid for RMNCH as part of a collaboration between academics, donors and other stakeholders, including the Countdown to 2030 and the Partnership for Maternal Newborn & Child Health.^9^ The Muskoka2 method was designed to enable the analysis of both donors’ fulfilment of their commitments and the patterns of distribution of aid for RMNCH across recipients in a way that was efficient, timely, accurate, predictable and transparent. Muskoka2 sought to retain the simplicity and transparency of the original Muskoka approach, which was developed by G8 donors, while incorporating eight innovations to improve accuracy and enable more granular, recipient-level analyses by drawing on the strengths of the earlier Countdown to 2015 approach.

Muskoka2 is an algorithm applied to aid datasets maintained by the Organisation for Economic Cooperation and Development (OECD). The OECD datasets reflect aid reported by donors themselves in a comparable format, covering all development sectors. While the donors classify their reported aid by sector and subsectoral purpose, the categories do not permit straightforward estimates of aid for RMNCH. Conceptual and technical choices are therefore necessary to identify the share of total aid (to all sectors) to count within estimates of aid for RMNCH.

Consistent with the conceptualisation of RMNCH within the Every Woman Every Child Global Strategy,^10^ the Muskoka2 method seeks to estimate the monetary value of aid directly contributing to improvements in RMNCH outcomes. It therefore includes the full monetary value of aid categorised by donors as being directed towards reproductive health and family planning and includes relevant shares of aid directed towards HIV, malaria and other infectious diseases; health systems and basic healthcare; the humanitarian and water and sanitation sectors; and general budget support. The shares of aid for HIV, malaria, tuberculosis and general budget support counted towards RMNCH vary between recipient countries and over time to account for differences in demography, epidemiology and health spending. Muskoka2 is therefore suitable for use in analyses of the distribution of aid across recipient countries. Muskoka2 produces separate estimates of aid for maternal and newborn health, aid for the health of children aged 1–59 months and aid for the reproductive health of non-pregnant women.

**Figure 1:**
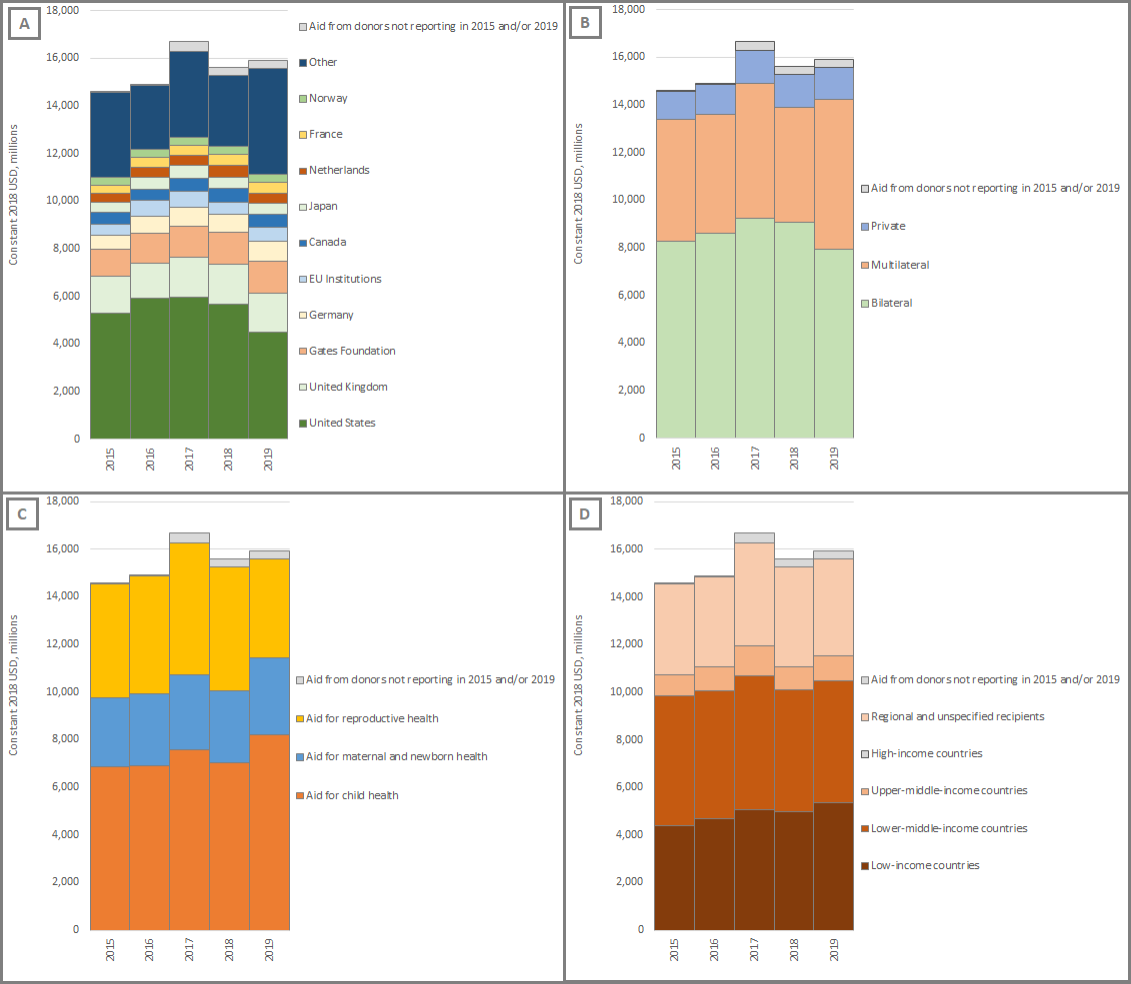
Trends in aid for RMNCH, 2015–2019. Values cited in the text are restricted to donors reporting in both 2015 and 2019 to avoid reporting bias in trends over time, as some donors did not report in all years. Panels show trends in aid for RMNCH: (A) disaggregated by donor, including both direct bilateral disbursements and core contributions to multilateral institutions from the top 10 largest donors over the time period and other bilateral and private donors; (B) disaggregated by type of donor deciding the purpose and recipient of the disbursement; (C) disaggregated by aid for child health, for maternal and newborn health and for the reproductive health of non-pregnant women; and (D) disaggregated by the World Bank country income group classification for recipient countries and regional and unspecified recipients. EU, European Union; RMNCH, reproductive, maternal, newborn and child health; USD, United States dollars.

We applied the Muskoka2 method to the most recent OECD datasets (January 2021 release).^11^ We included all disbursement data on official development assistance loans and grants and private development finance. To provide a picture of the aid landscape just before COVID-19 emerged, we present disbursements from the 48 donor countries, 35 multilateral institutions and 10 private donors reporting for both 2015 and 2019—that is, across the 5 years leading up to the pandemic (figure 1)—and focus on changes since 2017, the year for which estimates of RMNCH aid were last published.^9 12^

## Falling aid for RMNCH

Aid for RMNCH fell by 6% from $16.7 billion (constant 2018 US dollars) in 2017 to $15.6 billion in 2018, and only increased by 2% in 2019, to $15.9 billion. In the same period, total aid for all sectors increased from $262 billion in 2017 to $267 billion in 2018 (2%) and again by 2% in 2019, reaching $273 billion, indicating falling prioritisation of RMNCH.

The reduction in aid for RMNCH from 2017 to 2018 was largely driven by a collective decrease (−18%, −$643 million) in disbursements from smaller donors (outside the top 10, see figure 1) and decreases by the USA (−$287 million, −5%), European Union (−$198 million, −29%) and Japan (−$58 million, −11%). From 2018 to 2019, the USA further decreased its aid for RMNCH (−$1163 million, −20%)—mostly through reductions in support for HIV/AIDS—as did the Netherlands (−$77 million, −15%) and Canada (−$46 million, −8%). These 2018–2019 reductions were, however, offset by a 30% ($1459 million) increase in aid for RMNCH from multilateral institutions, notably UNICEF, GAVI, the Global Fund and United Nations Population Fund (UNFPA).

From 2017 to 2018, aid for child health, maternal and newborn health, and the reproductive health of non-pregnant women fell by 7%, 5% and 6%, respectively. Over the 2-year period from 2017 to 2019, however, aid for child health increased overall by 9% to $8.2 billion and aid for maternal and newborn health increased by 2% to $3.2 billion, whereas aid for the reproductive health of non-pregnant women fell by 25% to $4.2 billion. Low-income countries collectively received an increasing share of aid for RMNCH (34% in 2019), but the value of this aid fell from $5063 million in 2017 to $4965 million in 2018 (−2%), before rising by 8% to $5363 million in 2019.

## Challenges in tracking resource flows for RMNCH

These estimates are, of course, imperfect. Some donors, including China, do not report their aid to the OECD, so we may underestimate aid to some degree.^13^ Donors do not all report their aid entirely accurately, and there are substantial reporting delays; we will have to wait until early 2022 for the OECD to release sufficiently detailed disbursement data to estimate aid for RMNCH in 2020.

While the Muskoka2 method offers many advantages, it cannot estimate the contribution to RMNCH of individual projects and, as an algorithm, it inevitably involves simplifications and assumptions, which have been discussed in detail elsewhere.^9^ Muskoka2 does not produce disaggregated estimates of aid to family planning or newborn or adolescent health; key term searches and manual coding may be used to explore these and other areas within the data.

Two other approaches are used to track aid for RMNCH in quite different ways.^14^ One approach, the RMNCH policy marker, is implemented by donors themselves within the OECD’s aid activities database. Donors are asked to code each aid activity they report according to whether it fully, partially or does not support RMNCH. The approach is thus not designed to produce estimates of aid for RMNCH and donors report inconsistently, limiting its usefulness for aid tracking, despite the apparent advantages of individually categorising each aid activity.^14^ The Institute for Health Metrics and Evaluation (IHME) produces estimates of development assistance for health, which it disaggregates by health area, including reproductive and maternal health and neonatal and child health.^15^ IHME’s approach includes key term searches to categorise activities directed towards RMNCH, excluding aid directed towards infectious diseases, the health system or the humanitarian or water and sanitation sectors; it is applied to a combination of OECD disbursement data, other data sources and projections for more recent years.

None of these aid tracking methods can currently be used to analyse domestic financing for RMNCH, which is a crucial component of the overall RMNCH resource envelope. Assessment of whether reductions in aid are offset by increases in domestic financing would be valuable; however, estimates of domestic financing for RMNCH are only available for selected years for selected low-income and middle-income countries^16 17^ and are very resource and time intensive to produce.^18 19^ With the Countdown to 2030, we are working to develop a Muskoka2-style algorithm that can be used to track domestic allocations to RMNCH in a timely and transparent manner.

## Conclusion

While the Muskoka2 estimates published in 2020 painted a hopeful picture of a 10% increase in aid for RMNCH from 2016 to 2017,^9^ our updated findings, with two more years of data, are worrying. Our findings suggest that volatile and falling RMNCH investments may have rendered RMNCH systems more fragile in the 2 years preceding the COVID-19 pandemic. If World Bank predictions of falling overall global aid levels in 2021–2025^20^ are correct, health and RMNCH in particular would need to be increasingly prioritised just to maintain recent RMNCH aid levels. A slight increase in the US global health aid is expected in 2021–2022,^21^ but the U—the second largest bilateral donor—has announced substantial cuts to the health and humanitarian sectors.^22^ In both countries, new commitments to COVID-19 and ‘global health security’^21 22^ within stagnant or shrinking health aid budgets risk crowding out RMNCH at a time when more, not less investment is needed.

We present only the geographic and demographic destinations of aid; however, many other analyses are possible with these data. We therefore encourage everyone academics, advocates and policy makers—to explore and exploit the Muskoka2 aid for RMNCH dataset,^12^ as well as the wider OECD aid datasets.^11^ Future work should further explore measures of aid effectiveness, including the channels through which RMNCH funds are disbursed,^7 23^ and equity in the allocation and distribution of RMNCH aid.^16^ Such analyses are crucial to guide advocacy efforts to improve RMNCH outcomes, especially in low-income countries.

## Data Availability

Data are available in a public, open access repository: https://datacompass.lshtm.ac.uk/id/eprint/2272/.
